# Membrane Fusion Induced by Small Molecules and Ions

**DOI:** 10.1155/2011/528784

**Published:** 2011-05-04

**Authors:** Sutapa Mondal Roy, Munna Sarkar

**Affiliations:** Chemical Sciences Division, Saha Institute of Nuclear Physics, Sector 1, Block AF, Bidhannagar, Kolkata 700064, India

## Abstract

Membrane fusion is a key event in many biological processes. These processes are controlled by various fusogenic agents of which proteins and peptides from the principal group. The fusion process is characterized by three major steps, namely, inter membrane contact, lipid mixing forming the intermediate step, pore opening and finally mixing of inner contents of the cells/vesicles. These steps are governed by energy barriers, which need to be overcome to complete fusion. Structural reorganization of big molecules like proteins/peptides, supplies the required driving force to overcome the energy barrier of the different intermediate steps. Small molecules/ions do not share this advantage. Hence fusion induced by small molecules/ions is expected to be different from that induced by proteins/peptides. Although several reviews exist on membrane fusion, no recent review is devoted solely to small moleculs/ions induced membrane fusion. Here we intend to present, how a variety of small molecules/ions act as independent fusogens. The detailed mechanism of some are well understood but for many it is still an unanswered question. Clearer understanding of how a particular small molecule can control fusion will open up a vista to use these moleucles instead of proteins/peptides to induce fusion both in vivo and in vitro fusion processes.

## 1. Introduction

Membrane fusion is the process of merging of adjacent cells, vesicles, or liposomes to mix their inner contents and to form a large fused cell, vesicle, or liposome [1]. It is an integral process of many biological events starting from gamete formation by fertilization [2, 3], viral infection of host cells [4, 5], endocrine hormone secretion [6], and neuronal signaling [7]. Thus, membrane fusion is essential, not only for the initiation of life but also to carry forward it successfully without any difficulties. The process of membrane fusion is extremely important for study, in order to find out a way to control it and to make the process more useful in various in vitro biochemical processes and in biotechnology [8]. 

**Figure 1:**
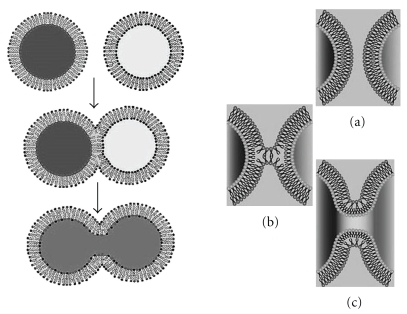
Basic Steps of membrane fusion. (a) Membrane contact, (b) outer leaflet lipid mixing to form the hemifused state, and (c) inner leaflet lipid mixing and pore formation and content mixing.

The process of membrane fusion varies widely in different systems. For example, fusion of yeast vacuoles (dimension-micron level) occur through an area of contact, which is almost 10000-fold larger compared to that during exocytosis of synaptic vesicles. Whereas, the vacuoles take minutes to undergo fusion, the synaptic vesicles take milliseconds, which means *∼*10000-fold shorter times than yeast vacuoles [9]. Fusion both in vivo and in vitro is usually induced by external agents called fusogens. The most common fusogens are large molecules like proteins and peptides. There are also other types of fusogens which will be detailed later in the text. Depending on the nature of the fusogens, the exact mechanism varies. By “mechanism,” we mean the way a fusogenic agent will induce the fusion process. Despite the diversities in the mechanism, the process is characterized by similar basic steps in all kinds of membrane fusion (Figure 1). In order to fuse two membranes, they must first be brought together such that their surfaces become closely apposed. This requires removal of aqueous environment associated with the polar head groups and is expected to be one of the most energetically demanding process [10]. This is followed by a local disruption of the organized bilayer which results in fusion of outer leaflet of each membrane forming the hemifused, often “stalk-like” intermediate. Next, reorganization of the inner lipid leaflet results in pore opening and mixing of inner aqueous contents to complete the fusion process. Each intermediate state of the fusion process is characterized by their specific conformational energy which is the associated potential energy of the state. The difference in the conformational energy between state 2 and state 1 provides the potential energy barrier. This needs to be overcome for the fusion to proceed. A fusogen provides this energy to overcome the barrier which is translated into the required kinetic energy that drives the membrane from one intermediate step to the next by structural reorganization of the lipid molecules in the bilayer. If the amount of energy supplied by the fusogens is not enough to strike the correct “structure energy balance,” that is, the energy supplied is not adequate to form the correct structure of the next intermediate, the fusion process will not be able to proceed further.

The basic steps of membrane fusion are detailed below.

### 1.1. Vesicle Contact

It is *van der Waal's *attractive interaction that brings together the two membranes to undergo fusion. The extent of this attractive force has been estimated according to the Derjaguin-Landau-Verway-Overbeek (DLVO) theory [11]. Besides van der Waal's attractive force, there are hydration, electrostatic, and steric forces that produce strong repulsive force adequate enough to prevent the close contact of the approaching membranes [12]. The interplay of these two types of opposing forces makes a vesicle suspension stable at physiologically relevant conditions. Generally, the intercellular or intervesicular distance of *∼*2 nm is mostly due to the prevailing excessive hydration repulsion that acts in between two approaching membranes [13].

There are several ways to initiate this approach to make a close contact between two fusing vesicles. Diminishing the intermembrane hydration repulsion is one of the methods to initiate membrane contact [13]. The hydrophilic, aqueous environment between two approaching hydrophobic bilayers is the origin of the hydration repulsion. In fact, lowering of the number of water binding sites or charge on the membranes decreases the lipid bilayer repulsion, which facilitates close contact of the membranes [14]. Different fusogenic agents have different mechanisms to diminish this hydration repulsion. These fusogens can act either by decreasing bilayer surface charge density, polarity or by increasing hydrophobicity of the intermembrane hydrophilic region by dehydrating the intermediate environment as in case of poly (ethylene glycol) [15, 16]. An estimated >100 atm pressure is required to mix or merge the outer leaflet of contacting membranes [17], which is very high. To minimize the work required for lipid merger, initial contact proceeds is expected to through small, local point of contact. This leads to the participation of minimum number of lipid molecules during formation of the fusion intermediates. Thus, merging of lipid membranes becomes a very site-restricted process, acting in some cases within a very small area of approximately 10 nm^2^ [18]. 

### 1.2. Vesicle Merging

**Figure 2:**
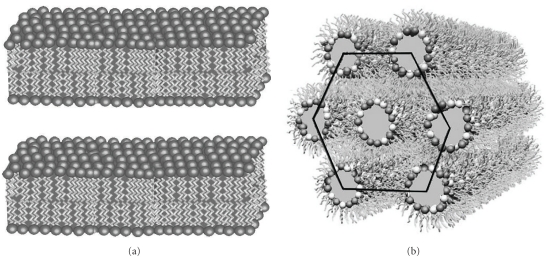
Different phases of lipid: (a) lamellar (L) phase and (b) inverted hexagonal (H_II_) phase.

After the close contact of the two approaching membranes or lipid bilayers, a temporary disorder of the bilayer lipids in the contact region is required for vesicular merging. There are several ways to achieve this. The lipid composition of the vesicles plays a crucial role during the merging of membranes. According to a school of thought [19], during the merging of lipids, considerable amount of lipids undergo a transition from lamellar bilayer phase (L) to inverted hexagonal phase (H_II_) (Figure 2) [20] at the contact site. It has been proposed that, the transition from L → H_II_ is essential for the successful mixing of the outer bilayer of lipids to promote fusion. There are several inverted hexagonal phase forming lipids, namely, phosphatidylethanolamine (PE) [21] and cardiolipin [22] which influence the required structural/orientation change of the lipid molecules during merging. There are a number of molecules like drugs [23], surfactants [24], solvents [25], and metabolites [26] which can influence the L → H_II_ phase transition and thereby have the propensity to act as fusogens. 

Another factor that facilitates the merging of the lipids and thus promotes fusion is accumulation of defects or perturbations or fluctuations in the contact region. Defects can be introduced either by external agents like fusogens, or by alteration of some physical parameters. It is well known that temperature [27], membrane curvature [28], surface tension [27], and so forth, can incorporate stress and strain in the membranes which introduce defects. The effect of temperature generally maximizes near the phase transition temperature of the lipid bilayer where a minute change in temperature causes significant defect or fluctuations in the bilayer that is enough to induce fusion [27]. Surface tension, which represents the surface density of membrane surface free energy, is another physical parameter that is known to induce fusion. Increase in surface tension supplies the requisite energy to overcome the barriers of different steps of fusion to complete the process [16, 27]. 

Small lipid vesicles of diameter *∼*45 nm do not have sufficient number of lipid molecules for forming inverted hexagonal phase, yet they are known to fuse extensively [29]. The high curvature induces defects in membranes in such a way that the membranes get the required energy for fusion. The spontaneous curvature of the lipid molecules is a critical factor for lipid merging and intermediate formation. Spontaneous curvature of lipids means the curvature of the lipid monolayer achieved in the aqueous solutions in absence of any constraints. It is determined by the molecular structure of lipids within the monolayer. Spontaneous curvature, which is an inherent property of the lipid molecules, dictates the effective shape of the lipids in the bilayer [30, 31]. Lipids having positive spontaneous curvature lead to an inverted cone-shaped structure. Whereas, lipids having negative spontaneous curvature have cone-like shape. Lipids with cylindrical effective shape have zero spontaneous curvature [32]. Thus, the choice of specific lipid molecules can also control the process of fusion, by dictating the nature of the spontaneous curvature. It is now established that the stress and strain associated with high membrane curvature, provide enough perturbation and energy source for fusion [27].

### 1.3. Membrane Fusion Assays

Before going into the details of the use of various fusogenic agents, we will briefly describe how the process of fusion is monitored. Apart from imaging techniques, namely, transmission electron microscopy (TEM), scanning electron microscopy (SEM), freeze fracture microscopy, confocal microscopy, and fluorescence microscopy that can monitor fusion of large cells or vesicles, there are fluorescence assays which can monitor the fusion of vesicles having diameter as small as *∼*20 nm.

Fluorescence assays are used to monitor the initial step of fusion, that is, lipid mixing, and also the final step of membrane fusion, that is, the mixing of inner contents of the vesicles. Leakage of inner contents of vesicles through the membranes is a process which occurs spontaneously and continuously in the vesicles. Spontaneous leakage occurs simultaneously with content mixing and lipid mixing. Measurement of leakage is necessary to get a quantitative idea about the extent of content mixing and lipid mixing. Here, we describe the two principal assays mostly used in the literature, namely, lipid mixing assay and content mixing assay.

#### 1.3.1. Lipid Mixing Assay

Lipid mixing is the preliminary step of membrane fusion, where after the initial contact, the lipid molecules from both the leaflet of the bilayer mix together. This leads to the formation of the hemifusion state that often extends to form a stalk like intermediate. Most commonly, Forster Resonance Energy Transfer (FRET) between donor and acceptor molecules are used to monitor lipid mixing. The widely used probes are, donor: N-NBD-PE [N-(7-nitrobenz-2-oxa-1,3-diazol-4-yl)-1,2-dihexadecanoyl-sn-glycero-3-phosphoethanolamine, triammonium salt] and acceptor: N-Rh-PE [Lissamine rhodamine B-1,2-dihexadecanoyl-sn-glycero-3-phosphanolamine, triethylammonium salt] [33]. One set of vesicles are prepared by tagging them with both the donor and acceptor probes, where the distance between the donor and acceptor molecules are such that FRET occurs. These are mixed with another set of vesicles without any probe. After fusion of the two sets of vesicles, the optimum distance between the donor-acceptor pair increases. This increased distance between N-NBD-PE and N-Rh-PE decreases their FRET efficiency and hence the fluorescence of the donor, that is, N-NBD-PE increases. The increase in fluorescence of the donor or the decrease in fluorescence of the acceptor is monitored to calculate the percentage lipid mixing as a function of time [33, 34]. 

#### 1.3.2. Content Mixing Assay

Content mixing is the mixing of inner contents of the two fusing vesicles and is the final stage of membrane fusion. That is why the measurement of content mixing is essential to show the successful completion of the fusion process. Two types of assays are generally used to probe content mixing. One is the Tb/DPA assay where terbium chloride (TbCl_3_) and dipicolinic acid (DPA) are used. The other assay is the ANTS (Aminonaphthalene-trisulphonic acid)/DPX (p-Xylene bis(pyridinium) bromide) assay [35, 36]. 

The Tb/DPA assay is done at pH > 5.0 and is generally meant for vesicles of all sizes. In Tb/DPA assay, two sets of vesicles are prepared, one encapsulated with TbCl_3_ and another encapsulated with DPA. Though both Tb^3+^ and DPA are nonfluorescent in nature, their complex is highly fluorescent, and the fluorescence of the Tb^3+^-DPA complex is used to monitor content mixing [36]. Content mixing results in the formation of Tb^3+^-DPA complex which increases the fluorescence intensity. This is used to calculate percentage content mixing as a function of time. To avoid fluorescence from Tb^3+^-DPA complex leaking out of the vesicles, a powerful chelating ligand EDTA is added to the external buffer that breaks the Tb^3+^-DPA complex and makes a nonfluorescent complex Tb^3+^-EDTA [35].

The ANTS/DPX assay is done at low pH (pH < 5.0), where Tb/DPA assay is nonfunctional. Mostly large unilamellar vesicles (LUVs) fusion and giant unilamellar vesicles (GUVs) fusion can be monitored using this assay [36]. The ANTS/DPX assay is based on the collisional quenching of the ANTS fluorescence by the quencher DPX [37]. Two sets of vesicles are prepared, one encapsulated with fluorescent probe ANTS and the other encapsulated with fluorescence quencher DPX. Due to fusion, the inner contents of the vesicles get mixed together and the fluorescence of ANTS is quenched by DPX. The measurement of the decrease in fluorescence intensity gives the estimation of extent of content mixing [36, 38]. 

## 2. Fusion Induced by Different Fusogenic Agents 

Membrane fusion requires direct or indirect participation of different external agents or fusogens. There are various ways that a fusogenic agent can induce fusion. As has been mentioned before, a crucial structure-energy balance is required for every intermediate step of the fusion process. Lack of which may prevent the completion of the process or may cause the process to revert back to the initial unfused stage [39, 40]. There are various kinds of fusogenic agents available. Amongst them proteins and peptides are most common in biological systems. Large molecules like proteins and peptides can overcome the energy barrier of the intermediate steps of membrane fusion by reorganization of their structures which provide the driving force [41–46]. Hence, most fusion events both in vivo and in vitro are induced by proteins and peptides. Small molecules and ions, unlike proteins and peptides, cannot provide the necessary driving force by their structural reorganization to induce and complete membrane fusion.

Apart from proteins and peptides, there are lipids [47], small organic ligands [48], metal ions [49], polymers [50], and drugs [51] that can act as fusogens. The mechanism by which different fusogens induce fusion, varies widely. The principal aim of this review is to probe the literature status and the possible mode of action of small molecules or ions on membrane fusion. This is because membrane fusion induced by more popular big molecules, namely, proteins or peptides is a well-studied and reviewed field [32, 42]. Several recent reviews have been solely devoted to proteins- or peptides-induced membrane fusion, where the mechanism has been clearly elucidated. Reviews on membrane fusion induced by small molecules and ions are rare in the literature. Since the mechanism of fusion in this case is expected to be very different from those of big molecules as mentioned above, we feel that this review is timely. Understanding the different mechanisms by which small molecules and ions induce fusion, will allow the use of these agents to induce fusion in a controlled manner in in vitro biochemical processes necessary in biotechnology. 

### 2.1. Positive Ions

Amongst all the small molecules, cations were first identified to have membrane fusogenic property. A wide range of studies were done starting from early seventies to late eighties on cations-induced fusion. From almost two decades of work, two cations, namely, Ca^2+^ [52–58] and Mg^2+^ [16, 59, 60] were found, which can induce membrane fusion effectively. Other cations like Mn^2+^ [49], Zn^2+^ [61–63], La^3+^ [64], Sr^3+^ [16], and H^+^ [65] also have some potential to induce membrane fusion, but Ca^2+^ is the principal player. Ion binding to membranes, not only results in screening of electric charges on membrane surfaces, but also modifies surface polarity, which in turn alters the hydration-dependent intermembrane repulsion [66]. 

#### 2.1.1. Pure Ca^2+^-Induced Fusion

Being a cation, Ca^2+^ can induce the fusion of anionic phosphatidylserine (PS) vesicles or phosphatidylserine:phosphatidylcholine (PS : PC) mixed vesicles. The negative charges of the vesicles prevent the close approach of the pure PS. It is the total amount of positive charge bound to the surface and the ionic strength of the buffer solution [67] that participate in charge screening, which in turn promotes vesicle aggregation. Studies have shown that the onset of close apposition of bilayers and hence fusion, directly depend on the destabilization of the membranes, which is proportional to the number of Ca^2+^ bound to the membrane surface. Presence of Na^+^ in the buffer competes with Ca^2+^ binding to anionic membrane surface. As the concentration of Na^+^ increases in the solution, the value of Ca^2+^ bound per PS molecule is diminished, which inhibits the Ca^2+^-induced fusion [60]. Experiments have been done using small unilamellar vesicles (SUVs) of PS or PS : PC mixed lipids. Theoretical studies and Tb/DPA assay, as described earlier, are used to monitor the fusion process [60]. Ca^2+^-induced fusion of PS vesicles occurs at a threshold concentration of about 1 mM [68] when the number of Ca^2+^ bound per PS molecule at the membrane surface is 0.40 [69, 70]. The process of fusion increases significantly above this threshold concentration. For PS : PC mixed vesicles, since PC is neutral in nature, there is a decrease in surface charge density which results in lower number of Ca^2+^ associated per PS molecule. The threshold concentration of Ca^2+^ is therefore higher, 2 mM for 4:1 PS : PC mixed vesicles in presence of 100 mM Na^+^ solution, where the Ca^2+^ bound per PS molecule is 0.36. For 2 : 1 PS : PC mixed vesicles, the threshold concentration is reported to be 4-5 mM, where Ca^2+^ bound per PS molecule is 0.38 [67]. Thus, presence of critical binding ratio is required to initiate fusion in pure PS or PS : PC mixed vesicles. As the binding of Ca^2+^ per PS molecule exceeds the threshold value of 0.35–0.39, the fusion process is initiated.  Table 1 shows the binding ratio of Ca^2+^ per PS molecule available in the literature [67], which will help in finding out the optimum criteria for Ca^2+^-induced fusion under specific conditions. 

Mg^2+^ also functions in a very similar way as Ca^2+^. Since Mg^2+^ has lower binding constant with PS molecule compared to that of Ca^2+^ [70, 71], the effectiveness for fusion of PS vesicles is lower in case of Mg^2+^. The threshold concentration of Mg^2+^ in 2 : 1 PS : PC mixed vesicles is ~10 mM and in pure PS vesicles is ~6 mM [59].

**Figure 3:**
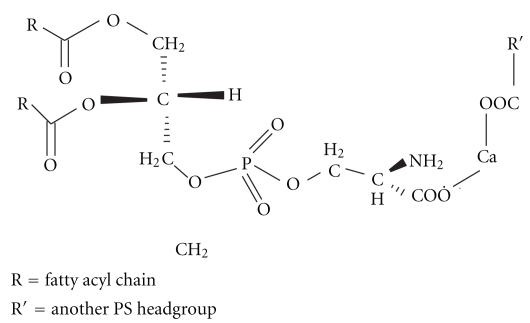
Binding of Ca^2+^ with PS headgroup.

Along with Ca^2+^, all other divalent cations are expected to follow a similar kind of mechanism of fusion. Although charge screening is necessary for the close association of the vesicles, but it is not sufficient to complete the fusion of PS containing vesicles. During fusion induced by Ca^2+^ or any other divalent cations, there is also an increase of membrane surface tension resulting in lateral compressibility and structural defects [16, 56]. This change in PS membrane surface is extremely necessary [49] for destabilization of the lipid bilayer along with surface charge screening by cations. Also, presence of Ca^2+^ or other divalent cations, the negative PS headgroups readily absorb the positive divalent ions, forming covalent bond through the carboxylate oxygen of the PS headgroup (Figure 3). This uptake of cations by the negatively charged sites of PS headgroups either cause the removal of structural water from the membrane surface [72], or cause the formation of “trans” “divalent cation-phosphatidylserine” complex. This brings the negatively charged PS headgroups close together; forcing the hydrocarbon phase of the membranes to face the hydrophobic phase [73, 74] thereby promoting the H_II_ phase formation that is conducive for membrane fusion.

#### 2.1.2. Ca^2+^-Induced Fusion in Presence of Sulfatide

**Figure 4:**
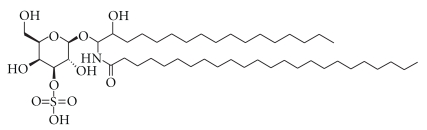
Sulfatide.

Other than PS vesicles, where electrostatic or charge-charge interaction plays a critical role during fusion, Ca^2+^ is also able to induce membrane fusion in vesicles containing lipids like PE and so forth. It has been found that 10 mM of Ca^2+^ enhances fusion of SUVs of dioleoylphosphatidylethanolamine (DOPE) containing less than 30 mole % sulfatides (Figure 4) [75]. The progress of fusion is monitored using two well-known assays, namely, NBD-PE/*N-*Rh-PE assay for lipid mixing and Tb/DPA assay for content mixing. PE has a natural tendency to fuse, but it is the negative charge of the sulphate (SO_4_^2−^) moiety of sulfatides that prevents the inverted hexagonal (H_II_) phase formation in the DOPE vesicles by interfacial hydration, and hence this leads to the stabilization of the DOPE-sulfatide SUVs [76, 77]. The binding of positively charged Ca^2+^ to the negatively charged sulfatides not only neutralizes the negative charge present on the membrane surface, but also reduces bound water, thereby dehydrating the membrane surface [78]. This results in the formation of nonlamellar intermediate as the PE molecules revert back to the H_II_ phase [79, 80]. Thus, fusion occurs following similar “destabilization of the membrane surface” mechanism as mentioned in case of pure Ca^ 2+^-induced fusion.

H^+^also functions in a similar manner by charge neutralization at low pH, which triggers dehydration and fusion. The DOPE-sulfatide vesicle fusion is also a pH-dependent phenomenon. At high concentration of sulfatides, that is, at sulfatide concentration >30 mol %, steric hindrance [81–83] of the sulfatide molecules on the membrane surface prevents the proton-induced or Ca^2+^-induced hydration-dehydration process. So, the fusion process is no longer pH-sensitive, and the concentration of Ca^2+^ has no direct effect. At such high concentration of sulfatide (>30 mole %), the fusion process decreases only with the increase in sulfatide concentration. 

#### 2.1.3. Ca^2+^-Induced Fusion in Presence of Other Fusogens

Ca^2+^ ions are also found to induce fusion in biomembranes like erythrocyte cell membranes [84]. In all cases where Ca^2+^ is found to induce fusion of vesicle containing lipids other than PS, different active fusogens (either lipid-soluble fusogens or water-soluble fusogens) are also required along with Ca^2+^. In these cases, Ca^2+^ only helps in making the event faster and more effective [85]. Both the lipid soluble-fusogens (oleoylglycerol, dioleoylglycerol, trioleoylglycerol, etc.) and water-soluble fusogens (polyethylene glycol, dimethyl sulphoxide, etc.) make the membranes more permeable [86]. The increase in permeability of the membranes facilitates the entry of the Ca^2+^ in the cell, and this increased partitioning of Ca^2+^ enhances the process of fusion. The entry of Ca^2+^ in erythrocyte cells is monitored by measuring the radioactivity of ^45^Ca^2+^ inside and outside of the cell. Though the mechanism of action of both the lipid-soluble and water-soluble fusogens is different, its actual aim is the same, that is, to increase the permeability of the membranes. Lipid-soluble fusogens influence the structure of the hydrocarbon chain and/or the polar headgroups [87], and water-soluble fusogens affect the membrane surface potential and/or hydration of the polar head groups [88] to increase the permeability of the bilayer. 

#### 2.1.4. Monovalent Cation-Induced Fusion

Among the monovalent cations, only H^+^ is found to induce membrane fusion. H^+^ induces aggregation and finally fusion of vesicles having negatively charged headgroups like PS SUVs [68, 89], PS LUVs [65], and other lipid vesicles like PE liposomes [90, 91], PS : PE mixed SUVs [79], PE-oleic acid mixed multilamellar vesicles [92], and PS-palmitoylhomocystein mixed SUVs [93]. 

#### 2.1.5. Trivalent Cation-Induced Fusion

**Figure 5:**
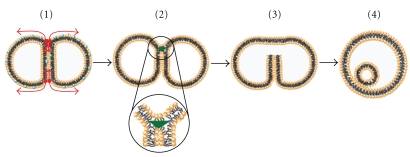
Schematic diagram of the partition breakage model. The arrows in (1) show the La^3+^-induced lateral compression pressure of the membranes, and the green triangle in (2) shows the interstitial hydrocarbon region where free energy of chain packing is very large. Adapted from reference [64].

A very new kind of mechanism, which is known as “partition breakage model” (Figure 5), where no stalk intermediate is formed, has been proposed in case of fusion of neutral GUVs induced by trivalent lanthanides, namely, La^3+^ or Gd^3+^ [64]. It has been observed that DOPE and dioleoylphosphatidylcholine (DOPC) mixed vesicle (30 DOPE : 70 DOPC) undergo membrane fusion on incorporation of 100 *μ*M of La^3+^. The process of fusion has been shown by phase contrast imaging. After the merging of the GUVs, the density of the incorporated La^3+^ is increased in the outer lipid layer (facing the buffer). Due to this, the lateral compression pressure of the outer monolayer increases. The presence of DOPE in the GUVs favors the formation of H_II_ (inverted hexagonal) phase, and La^3+^ stabilizes this H_II_ phase hence, the area of the outer monolayer is decreased. Thus, the chain packing, mostly at the edge of partition membrane, is destabilized causing the breakage of the membrane at the edge. Then, the area of this breakage site increases, and finally the partition membrane gets ruptured. This is a unique example where membrane fusion occurs directly starting from merging of the vesicles without the formation of any hemifusion (stalk-like) intermediate. Moreover, the partition membrane that has separated from the GUVs during fusion forms a tiny SUV and resides inside the fused vesicle. That is why the area of the membrane of the fused vesicle will be decreased due to the loss of partition membrane. 

### 2.2. Small Organic Molecule

#### 2.2.1. *n*-Hexyl Bromide

**Figure 6:**
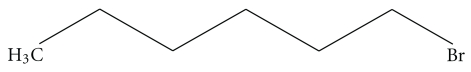
*n*-hexyl bromide.

During the early eighties, small molecules belonging to the* n*-alkyl bromide group [C_n_H_2n+1_Br; *n* ≥ 6] was found to act as fusogenic agents. Among them *n*-hexyl bromide (Figure 6; *n* = 6), octyl bromide (*n* = 8), and decyl bromide (*n* = 10) are known to have the desired fusogenic property [94]. These molecules can induce fusion at a very low molecule-to-lipid ratio (M : L) of 0.05 (for *n* = 6) [95], and they can induce cellular or subcellular membrane fusion [96] along with the fusion of biomimetics like PC-phosphatidic acid (PC : PA) mixed vesicles. Multilamellar vesicles (MLVs) of PC or PC : PA mixed lipids are used for experiments. Electron microscopy and freeze fracture microscopy images are shown to prove the successful completion of the fusion process [95]. Moreover, incorporation of very low amount of these organic molecules, for example, about 2.5 *μ*M of *n*-hexyl bromide/50 *μ*M lipid/mL does not alter the constitution, cell viability, or the ion transport properties of the membranes [95]. The fusion of vesicles was shown by electron microscopy images. A significant increase in the size of the vesicles due to the incorporation of *n*-hexyl bromide was shown [94, 95] as proof of fusion. 

In the early eighties, the small molecules that act as fusogenic agents were mostly metal ions. The only established mechanism, which was considered to be true at that time, was based on initial charge-charge interaction, charge neutralization, dehydration of membrane surface, and destabilization of the lipidic bilayer leading to fusion [49]. The mechanism followed by *n*-hexyl bromide was different from the mechanism that was common. That is why in the literature, the elucidation of mechanism of membrane fusion induced by *n*-hexyl bromide was mainly confined to showing that charge-charge interaction did not participate during the fusion process. No detailed mechanism of *n*-hexyl bromide-induced fusion was given. However, it was shown that fluidity of the membrane was a prerequisite for fusion.

#### 2.2.2. Short-Chain Alcohols—Ethanol

**Figure 7:**
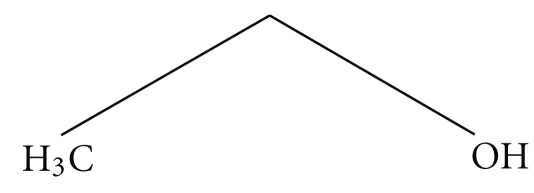
Ethanol.

Short-chain alcohols are found to induce the hemifusion process which finally leads the process to fusion. It is already known that general fusogens induce the formation of the H_II_ phase, which in turn stabilizes the hemifusion intermediate to promote fusion. Short-chain alcohols are expected to work differently. Alcohols, mostly methanol and ethanol (Figure 7), do not alter the curvature of the membrane bilayer. Both methanol and ethanol apparently support positive spontaneous curvature of lipid monolayers [97, 98]. That is why it is highly unlikely for them to induce the formation of the H_II_ phase. Researchers have found that short-chain alcohols like ethanol can induce the formation of the hemifusion or stalk intermediate in GUVs [99]. Moreover, ethanol is reported to induce the fusion of PC SUVs [100]. Imaging techniques like fluorescence microscopy and freeze fracture electron microscopy were used to monitor the fusion processes. Both the authors pointed that it was not the H_II_ phase formation but the destabilization of the outer membrane monolayer that led the process to complete fusion. During the formation of hemifusion or stalk intermediate, it was proposed that short-chain alcohols disrupted the outer leaflet of lipid layer to cause the local breakage of the monolayer to induce the stalk formation [99]. During stalk formation, the hydrophobic voids are created between the bilayer leaflets [101]. It was proposed that ethanol affected the lipid chain packing in the void region to decrease the energy of the hydrophobic voids to stabilize the stalk intermediate [102]. 

Moreover, a very high concentration of ethanol was proposed to form interdigitated state in the contacting region of the membranes. Interdigitated state formation led the hydrophobic acyl chain terminal to be exposed on the surface of the membranes, which increased the hydrophobic interaction between two contacting interdigitated bilayers. This resulted in the high stability of the aggregated lipids, which led the process to lipid mixing and finally to successful content mixing [100]. 

### 2.3. Plant Hormone—Abscisic Acid

**Figure 8:**
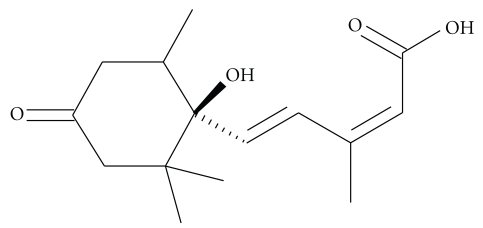
Abscisic Acid.

In the early nineties, another small molecule was found that can act as an effective fusogen, namely, a plant hormone, abscisic acid (ABA; Figure 8). It was found to promote fusion of vesicles consisting of a mixture of 9 : 1 PC : PE lipids at an ABA molecule-to-lipid ratio (M : L ratio) of 0.125 [103].

Though pure PC vesicles did not undergo fusion in presence of ABA, but at 9 : 1 PC : PE, vesicles start to fuse at an M : L ratio of 0.125 and the process of fusion increases as the M : L ratio increase from 0.125 to 0.50. Moreover, the fusion process also increased with the increase in the PE concentration in the mixed vesicles. All the fusion experiments were done using MLVs of dimyristoylphosphatidylcholine-dimyristoylphosphatidylethanolamine (DMPC/DMPE) mixed lipids at pH 5.0. ANTS/DPX assay was used to probe the mixing of the inner contents, and the NBD-PE/N-Rh-PE assay was used to probe the lipid mixing [103]. From these findings, it was evident that the presence of PE in the vesicles was essential for the initiation of fusion induced by ABA. PE is an intrinsically fusogenic lipid due to its capability of forming of H_II_ phase [104]. That is why PC-PE mixed vesicles are considered to be inherently unstable and capable of undergoing fusion spontaneously. Incorporation of PE in PC vesicles creates various sites of defects or perturbations in the membrane bilayer [105]. Mostly, the mismatch in headgroup size of the two phospholipids results in packing defects. It has been shown that ABA acts in these small regions of membrane defects. Since the working temperature is above the transition temperature of PC, but below that of PE, there are regions of interface between gel and liquid crystalline domains. ABA also occupies these interfaces [106]. After the two approaching membrane overcome the electrostatic, van der Waal's and hydration repulsion, a transient destabilization occur at the region of contact of the two membranes [107] which is enhanced by ABA. This enhanced destabilization causes a local dehydration, making the H_II_ phase formation a stable process and the bilayer structure of the membranes gets disrupted [108]. Finally, the size of the defect site is elongated and thus membrane permeability is also increased. This leads to aggregation of the lipids and to successful completion of fusion. 

### 2.4. Small Drug Molecules

#### 2.4.1. Halothane-Induced Fusion

**Figure 9:**
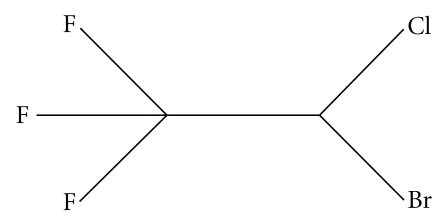
Halothane.

Halothane (Figure 9) is a small drug molecule that can act as an anesthetic [51]. Two-photon fluorescence correlation spectroscopy was used to show that LUVs of DOPC undergo fusion in presence of halothane. However, fusion can occur only at very high drug-to-lipid ratio (D : L) of 10.

Two-photon fluorescence correlation spectroscopy and ANTS/DPX assay for content mixing were done to probe the process of fusion. Halothane was heterogeneously incorporated in the bilayer just below the head group region of the lipids [109, 110]. This incorporation of halothane in the lipid bilayer causes the increase in the interlipid spacing. With increase in lipid spacing, random collision between vesicles become less elastic because lipid headgroups from each vesicles may interpenetrate which promotes the aggregation. Also, the void-filling effect of halothane would lower the energy of intermediate stalk formation [111], facilitating the fusion. 

#### 2.4.2. Oxicam NSAIDs-Induced Fusion

**Figure 10:**
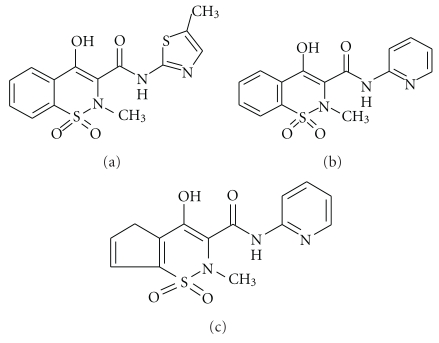
Oxicam NSAIDs: meloxicam (a), piroxicam (b) and tenoxicam (c).

The most recent discovery in the series of small molecule fusogens are the painkillers belonging to the oxicam groups of nonsteroidal anti-inflammatory drugs (NSAIDs). These drugs were originally developed to combat pain and inflammation but have been shown to have other functions as chemopreventive and chemosupressive agents [112–114]. Three drugs belonging to the oxicam groups of NSAIDs, namely, meloxicam, piroxicam, and tenoxicam (Figure 10) are found to act as membrane fusogens [115]. They are effective fusogens not only for membrane mimetics like lipid vesicles, but also for biomembranes like mitochondrial membrane. One of the drugs, piroxicam, was found to permeabilize mitochondrial membrane in V79 Chinese hamster lung fibroblast, at physiological concentration, leading to the release of cytochrome C in the cytosol that in turn signalled the downstream proapoptotic caspase-3 [116]. 

A closer look showed that membrane fusion is not a property of piroxicam alone but is also shared by two other oxicam painkillers, namely, meloxicam and tenoxicam [115]. The three drugs can induce membrane fusion at physiologically relevant concentration [117] whereas NSAIDs belonging to other chemical groups do not share this property.

There lies an immense potential for application of these drugs as inducers of membrane fusion. Understanding the mechanistic details of these NSAIDs-induced fusion will allow the use of these painkillers to fuse membranes in a controlled manner as is necessary in many biochemical/biotechnological processes. To identify and parse the effect of different physical and chemical parameters of both the participating drugs and lipids, simple membrane mimetics like SUVs, formed from phospholipid DMPC, were used. The kinetics of lipid mixing, content mixing, and leakage were followed by standard fluorescence assays, namely, NBD-PE/N-Rh-PE assay for lipid mixing and Tb/DPA assay for content mixing. These were coupled with direct imaging of fused vesicles with TEM. Temperature-dependent studies and concentration-dependent studies showed that, unlike the other small molecules, these drugs can induce fusion at a very low drug-to-lipid ratio of 0.018. The lipid mixing and content mixing are two sequential events, and the activation energy of both processes is lowered compared to smaller diameter vesicles, which promotes fusion. Effects of low concentration of cholesterol (<10 mol%) have also been studied in DMPC SUVs above the sol-gel transition of DMPC. It is known that under these conditions, there is an increase in orientational order of the lipids as well as an increase in headgroup spacing. The effect of these two membrane parameters counteract on the fusion process. The effect of orientational order dominates in case of meloxicam and piroxicam, leading to a decrease in the overall fusion process, but in case of tenoxicam, increase in headgroup spacing increases partitioning of the drug which nullifies the effect of orientational order [118].

The fact that these drugs are effective fusogens at such low drug-to-lipid ratio is a unique observation, when one considers that SUVs of DMPC are used. Phospholipids with PC headgroup are not known to have intrinsic tendency to fuse like PE headgroups [103]. Besides, SUVs having 50–60 nm diameter as used in this case do not have enough material to form the inverted hexagonal phase (H_II_) that promotes fusion [27, 119]. It should be mentioned that carefully designed controls in absence of the drugs showed that no fusion was taking place within the experimental time frame. This ruled out the fact that the high curvature of the SUV could induce spontaneous fusion. It is known that introduction of defects or membrane perturbation, either by external organic molecules or by changing physical parameters, can promote fusion [27]. In a previous work by the authors, where interaction of these three oxicam NSAIDs with DMPC monolayer was studied at air water interphase, it was found that in the same concentration range, the drugs were capable of perturbing the monolayer such that the monolayer plane was no longer well defined [120]. It is the perturbing ability of the drugs at such a low concentration range that can be one possible reason why these drugs are effective membrane fusogens.

#### 2.4.3. Chlorpromazine-Induced Fusion

**Figure 11:**
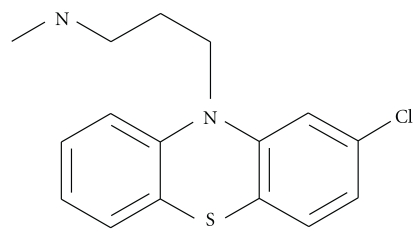
Chlorpromazine.

Chlorpromazine (CPZ; Figure 11) is a prototypical phenothiazine antipsychotic drug. CPZ can induce fusion of human red blood cells and viral envelopes without the aid of fusogenic proteins. However, this is achieved only when CPZ forms micelle-like aggregates [121–123]. Transmission electron microscopy (TEM), scanning electron microscopy (SEM), and so forth are used to have the images of fused vesicles. It has been found that at a concentration (2 mM) which is slightly less than the critical miceller concentration (CMC = 4 mM) of CPZ, it is able to fuse human RBC at pH higher than 6.8. These molecules act in the protein-free region of the membranes and cause fusion in the nonprotein site of the membranes [122].

Electron micrographs [124, 125] clearly showed that microdroplets of chlorpromazine adhere to the membrane surface of human RBC to form fused bodies. However, it is not established whether it is the microdroplets or smaller micelles that initiate the fusion process. CPZ has very good affinity for the RBC membranes, and it is intercalated inside the cytoplasmic half of the membrane bilayer. This results in the expansion of the inner monolayer of the RBC membrane, which destabilizes the bilayer that leads the process of fusion to successful completion [126, 127].

## 3. Concluding Remarks

**Figure 12:**
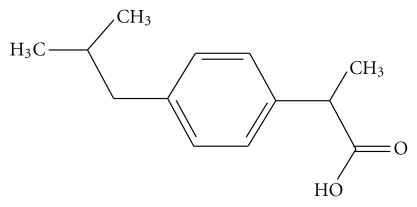
Ibuprofen.

The process of membrane fusion mediated by proteins/peptides is very different from that mediated by small molecules and ions. As we have already discussed, the basic difference that exists between these two types of fusion lies in their difference in the process of harnessing the energy required for overcoming the different intermediate steps of the fusion process. For protein/peptide-induced fusion, this energy is provided by the structural reorganization of the big molecules. Small molecules or ions do not have this advantage, as their structural reorganization is not expected to supply the required driving force. The mechanism for the process of fusion induced by small molecules/ions varies to some extent depending on the nature of the fusogen, though the basic steps of the fusion process remain similar. In general, small molecules get partitioned inside the membrane (or hydrophobic lipid phase) and cause a destabilization of the lipid bilayer. This destabilization manifests in membrane perturbation in different ways, depending on the nature of the small molecules. This perturbation may increase the permeability of the membranes, may make the collision between the vesicles more sticky, may increase the surface tension, or may even help in forming the inverted hexagonal phase (H_II_) formation. Any of these changes will help in inducing fusion. In case of ion-induced fusion, the primary effect is dependent on charge screening, where charge neutralization of the anionic lipid head groups by the cations promotes aggregation of the lipid vesicles and finally fusion occurs. However, in many cases, charge screening is found to be necessary but not a sufficient condition for the complete fusion. Additional factors like change in surface tension, surface hydration, and so forth, participate along with charge screening to lead the fusion to completion. This mechanism is different from the mechanism of small molecule-induced fusion as mentioned above.  Table 2, shows the threshold concentration of small molecules that is required to trigger membrane fusion. This has been compared with their dimension. Significant correlation exists between the concentration of the small molecules required for destabilization of the membrane bilayer, with their dimension. Even though there exists a correlation between molecular dimension and concentration of destabilization, it is not the only decisive factor. Molecules having similar dimension like ibuprofen (Figure 12) do not show fusion [115]. 

This is because it perturbs the membrane bilayer so much that leakage predominates in such way that fusion cannot occur. For a small molecule to act as an effective membrane fusogen, not only the dimension but also the chemical nature should be such that the lipid bilayer perturbation should not result in overwhelming leakage that prevents fusion.

There exists a gap in our knowledge in understanding the mechanism of how different small molecules and ions can induce membrane fusion and how the nature of these fusogens will dictate the process. A lot of work needs to be done to bridge this gap in our knowledge which is necessary to replace proteins and peptides by small fusogens for controlling fusion both in vivo and in vitro systems which will make the process more cost effective and easy to handle.

## Figures and Tables

**Table 1:** Calculated amount of Ca^2+^ bound per PS in mixed PS/PC membranes [60].

Vesicle composition (PS : PC)	Calculated tightly bound Ca^2+^ at various Ca^2+^ concentrations (in mM)						Na^+^ bulk concentration (in mM)
	0.7	1.0	2.0	3.0	5.0	10.0	
Pure PS	0.44	0.44	0.44	0.44	0.45	0.45	5
4 : 1	0.43	0.43	0.44	0.44	0.44	0.44	5
2 : 1	0.42	0.43	0.44	0.44	0.44	0.44	5
3 : 2	0.41	0.41	0.42	0.42	0.43	0.43	5
1 : 1	0.40	0.40	0.41	0.42	0.43	0.43	5
2 : 3	0.38	0.39	0.40	0.40	0.41	0.42	5
1 : 4	0.32	0.33	0.35	0.36	0.37	0.38	5

Pure PS	0.32	0.35	0.38	0.39	0.41	0.43	100
4 : 1	0.30	0.33	0.36	0.38	0.40	0.42	100
2 : 1	0.28	0.32	0.35	0.37	0.39	0.41	100
3 : 2	0.27	0.31	0.34	0.36	0.38	0.40	100
1 : 1	0.25	0.29	0.33	0.35	0.37	0.39	100
2 : 3	0.23	0.26	0.31	0.33	0.35	0.38	100
1 : 4	0.15	0.19	0.24	0.26	0.30	0.34	100

Pure PS	0.11	0.16	0.21	0.25	0.29	0.34	500
4 : 1	0.10	0.14	0.20	0.23	0.27	0.33	500
2 : 1	0.09	0.12	0.18	0.22	0.26	0.32	500
3 : 2	0.08	0.12	0.17	0.21	0.25	0.31	500
1 : 1	0.07	0.11	0.16	0.19	0.24	0.30	500
2 : 3	0.06	0.09	0.14	0.18	0.22	0.28	500
1 : 4	0.04	0.06	0.10	0.13	0.18	0.25	500

**Table 2:** Relation of the fusogenic molecules with its threshold concentration and size. For the determination of the length and width of the molecules, all the geometries are minimized using a semiempirical level of theory, Austin Model 1 (AM1) using GAUSSIAN 03 [128]. AM1 is based on a modified neglect of differential overlap (MNDO) approximation [129].

Molecules	Length (in nm)	Width (in nm)	Molecule-to-lipid ratio at threshold concentration	Vesicle size
Abscisic acid	1.04	0.68	0.125	MLV (*∼*1000 nm)
*n*-hexyl bromide	0.88	0.25	0.050	SUV (30–40 nm)
Ethanol	0.40	0.23		
Halothane	0.42	0.30	10.0	LUV (*∼*500 nm)
Meloxicam	1.43	0.60	0.018	SUV (50–60 nm)
Piroxicam	1.38	0.61	0.018	SUV (50–60 nm)
Tenoxicam	1.23	0.54	0.018	SUV (50–60 nm)
Chlorpromazine	1.06	1.01		6000–8000 nm
